# Lateral Posterior Method for Depth Correction while Using the Gates Protocol for GFR Estimation: Is it Comparable to the Gold Standard GFR Estimation by Plasma Sampling?

**DOI:** 10.1055/s-0044-1787100

**Published:** 2024-05-17

**Authors:** Shefali Madhur Gokhale

**Affiliations:** 1 Department of Nuclear Medicine, Sadhu Vaswani Missions Medical Complex, Pune, Maharashtra, India

**Keywords:** depth correction, GFR, Gates protocol, lateral posterior method, GFR by plasma sampling

## Abstract

**Background** Glomerular filtration rate (GFR) estimation by Gates protocol using the gamma camera for diethylenetriaminepentaacetic acid (DTPA) dynamic renography has not compared well with the gold standard GFR by plasma sampling method. This is because depth of the kidneys is generally not considered. Our aim was to study whether manual depth correction using the skin to middle of kidney distance in lateral view and posterior aspect-lateral posterior method would reduce the bias in the Gates GFR as compared with the gold standard.

**Materials and Methods** Retrospective study of 27 adult prospective renal donors who underwent GFR by plasma sampling and DTPA dynamic renography at Inlaks and Budhrani Hospital, Pune, Maharashtra, India between January 2022 and April 2023. The entire data was statistically analyzed using Statistical Package for Social Sciences (SPSS ver 21.0, IBM Corporation, United States) for MS Windows.

**Results** There is no significant agreement between plasma sampling versus gamma camera method and plasma sampling versus lateral posterior method for depth correction for GFR measurements; however, the evidence of systemic bias is lower for the gamma camera method compared with the lateral posterior method for depth correction as against the plasma sampling method.

**Conclusion** The lateral posterior method for depth correction while using the gamma camera-based Gates protocol is not a reliable method for depth correction in the western Indian adult population with preserved renal function.

## Introduction

The gold standard for measuring glomerular filtration rate (GFR) is studying the inulin clearance of the patient. 1 However, this is a cumbersome method and the cost and effort involved in procuring inulin is high. Various formulae have been used for estimation of estimated GFR (eGFR). In the western Indian donor population, Modification of Diet in Renal Disease equation has been found to be the most precise equation. 2 However, this does not give us an estimate of individual kidney GFR. This is where the gamma camera-based Gates method of estimating GFR came to have a role. But this too had significant deviations from the gold standard. It was opined that the gamma camera method did not take in to account the depth of the kidneys, which could be a reason for deviation from gold standard. Several methodologies were used for the calculation of depth of the kidneys. Anatomical modalities like computerized tomography (CT) kidneys, ureters, and bladder and ultrasound abdo-pelvis have been used for depth calculation. Various formulae like the Tonnesen's, Itoh's, and Taylor's were used for renal depth calculation. 3 Lateral views of the lumbar region after renal scintigraphy were acquired for renal depth calculation at our center. The GFR values thus obtained were compared with the gold standard.

eGFR calculated using serum creatinine/cystatin C using validated equations is the most widely used method of GFR estimation clinically and in epidemiologic research. Some of these formulae consider the race of the individual, thus introducing a race bias. 4 Most of these equations have been validated by comparison with GFR estimated by plasma clearance. 2 4 In view of the above and the availability of the gold standard method of GFR estimation by plasma sampling at our center, we chose this methodology for comparison rather than the widely used eGFR equations.

## Materials and Methods

This was a retrospective study from January 2022 to April 2023 at Inlaks and Budhrani Hospital, Sadhu Vaswani Mission's Medical Complex, Pune, Maharashtra, India. The patients considered were prospective renal donors.

Inclusion criteria were:

1. Greater than 18 years old
2. Had undergone GFR by plasma sampling, diethylenetriaminepentaacetic acid (DTPA) renal scan, and lateral views of the lumbar region
3. None of the patients had underlying hydronephrosis, renal calculi, renal tumors, and congenital renal anomalies

Written informed consent was obtained from all the renal donors.

**Fig. 1:**
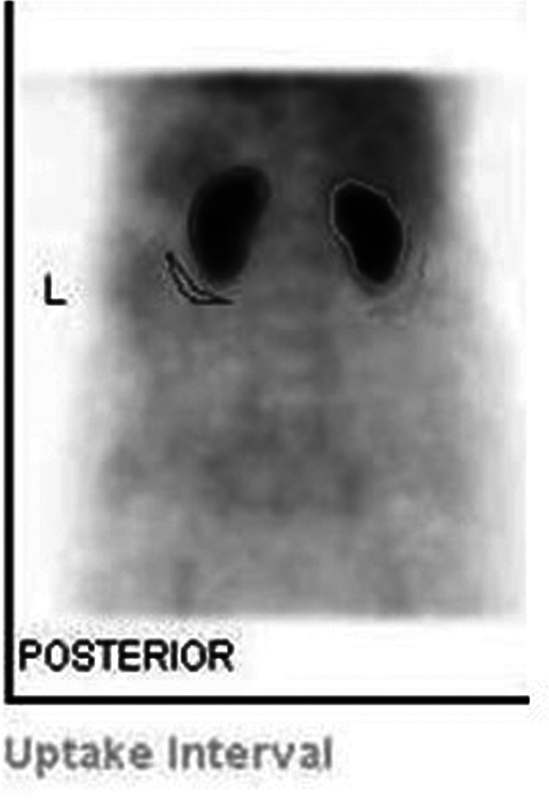
Regions of interest around each kidney and identical background regions of interest placed manually.

**Fig. 2:**
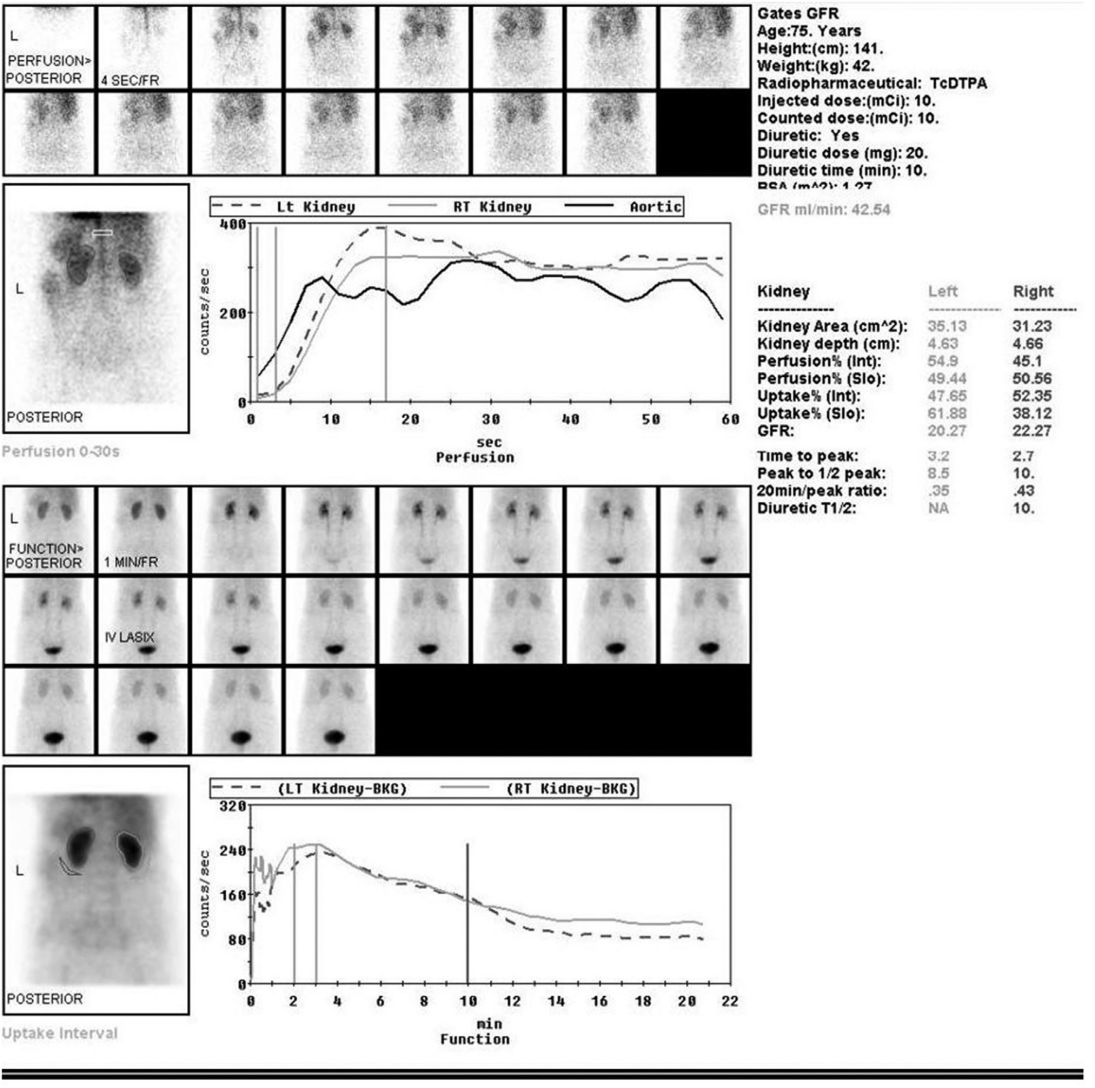
Glomerular filtration rate (GFR) calculated by inbuilt Gates formula after renal processing and relevant entries.

All patients were hydrated with 1 L of water for 10 minutes prior to the study. Age, gender, height, and weight of the patient were recorded in the system. Full syringe counts were recorded. A dose of 10 mCi Tc-99m DTPA was administered intravenously and a 20-minute DTPA renal scan was acquired in a 128 × 128 matrix using a GE Infinia Hawkeye 4 single photon emission CT-CT system. Renogram processing was done by placing regions of interest around each kidney manually. Identical background regions of interest were also placed manually ( Fig. 1 ). GFR calculated by the inbuilt Gates formula was generated by the system after relevant entries in to the system were made ( Fig. 2 ). Right and left lateral views of the lumbar region were acquired at delayed 1 hour.

The distance from the skin to the kidney center in the lateral view in its posterior aspect was chosen as the renal depth—lateral posterior method for depth correction. The values thus obtained were entered in to the system and the GFR thus generated was made note of. Blood samples were withdrawn 2, 3, and 4 hours postinjection of 10 mCi Tc99m DTPA. The GFR protocol of the Nuclear Medicine Procedure Manual of the Ottawa Hospital, Ottawa, Canada was used for estimation of GFR by plasma sampling by the slope-intercept method. 2

## Statistical Methods

The data on categorical variables is shown as *n* (% of cases) and the data on continuous variables is presented as mean and standard deviation (SD). The statistical significance of paired differences of means of GFR by each method (with reference to plasma sampling as a gold standard) is tested using paired *t* -test. The underlying normality assumption was tested before subjecting the study variables to the paired *t* -test. Intraclass correlation (ICC) analysis is performed to study the statistical reliability between the measurements separately by each method. Bland–Altman's method is used to study the extent of agreement between the two measurements (with reference to plasma sampling as a gold standard). Linear regression analysis is used as a part of Bland–Altman's methodology to test the statistical significance of the extent of bias (difference) present between the two measurements. All results are shown in tabular as well as graphical format to visualize the statistically significant difference more clearly. In the entire study, *p* -values less than 0.05 are considered to be statistically significant. The entire data are statistically analyzed using Statistical Package for Social Sciences (SPSS ver 21.0, IBM Corporation, United States) for MS Windows.

## Results

**Fig. 3:**
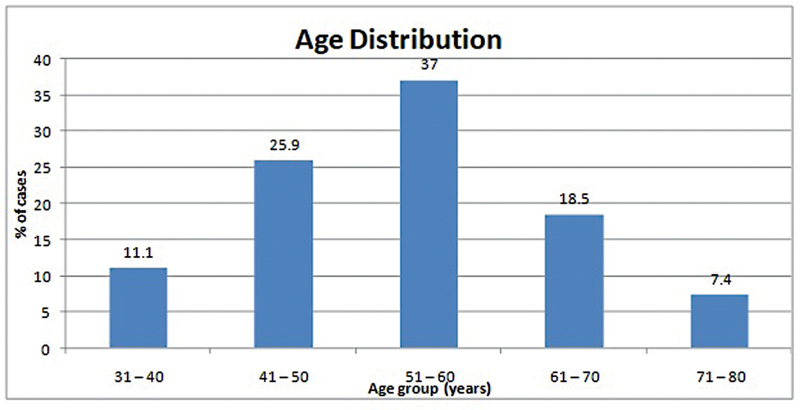
Age distribution of cases studied.

A total of 27 cases were studied. Three cases (11.1%) had age between 31 and 40 years, 7 cases (25.9%) had age between 41 and 50 years, 10 cases (37.0%) had age between 51 and 60 years, 5 cases (18.5%) had age between 61 and 70 years, and 2 cases (7.4%) had age between 71 and 80 years in the study group ( Fig. 3 , Table 1 ).

**Table 1:** Age distribution of cases studied

Age group (y)	No. of cases	% of cases
31–40	3	11.1
41–50	7	25.9
51–60	10	37.0
61–70	5	18.5
71–80	2	7.4
Total	27	100.0

The mean ± SD of the age in the entire study group was 53.63 ± 10.67 years and the minimum–maximum age range was 31 to 76 years. Twelve cases (44.4%) were male, and 15 cases (55.6%) were female. The male-to-female sex ratio was 0.80:1.00 in the study group.

The mean GFR by plasma sampling method was 94.81 ± 28.02 mL/min. The mean GFR by gamma camera method and lateral posterior depth (LPD) correction applied to the gamma camera method was 77.67 ± 18.97 and 63.09 ± 15.30 mL/min, respectively ( Table 2 ).

**Table 2:** Descriptive statistics of GFRs calculated by various methods

	GFR (mL/min)				
Method	Mean	SD	Median	Min	Max
Plasma sampling	94.81	28.02	97.00	42.00	145.00
Gamma camera	77.67	18.97	76.36	42.02	124.86
Lateral posterior depth	63.09	15.30	60.30	27.99	95.05

Abbreviations: GFR, glomerular filtration rate; SD, standard deviation.

**Fig. 4:**
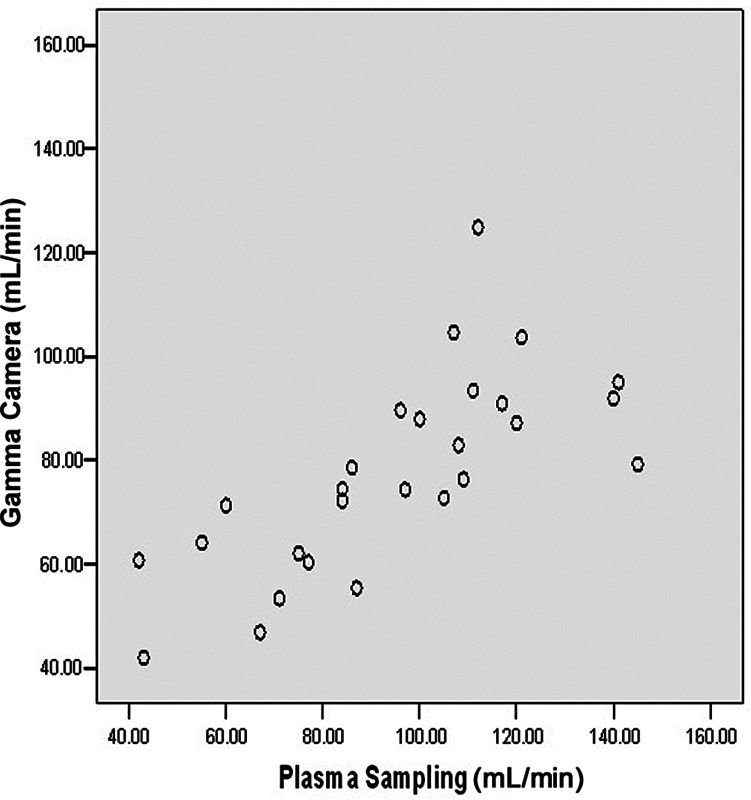
Scatter diagram showing correlation between glomerular filtration rates (GFRs) by gamma camera method versus plasma sampling.

**Fig. 5:**
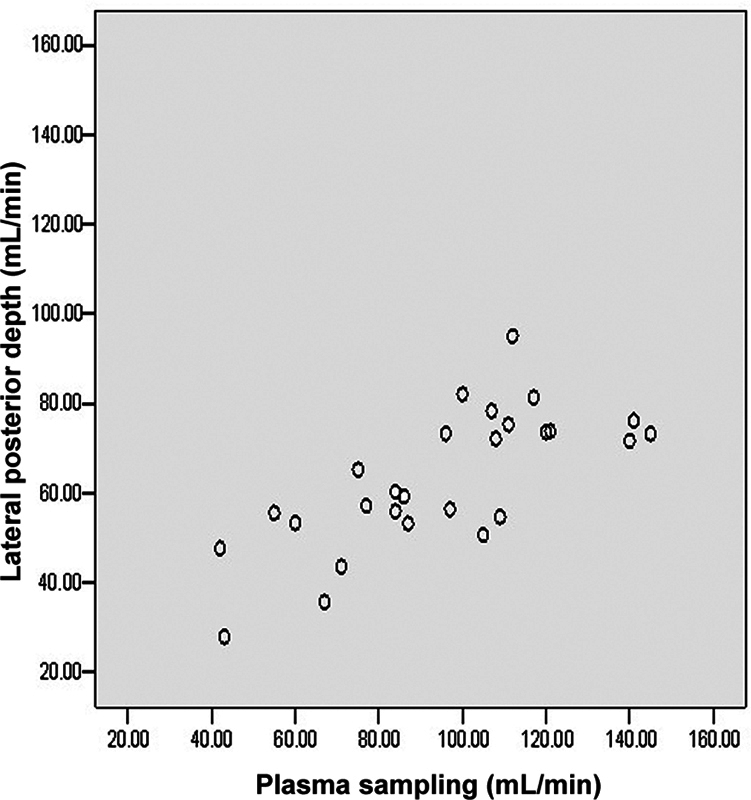
Scatter diagram showing correlation between glomerular filtration rates (GFRs) by lateral posterior depth method versus plasma sampling.

The mean GFR by plasma sampling is significantly higher compared with mean GFR by gamma camera ( *p* -value < 0.05) and GFR by lateral posterior method depth correction ( *p* -value < 0.05). GFR is significantly underestimated by the gamma camera method compared with the plasma sampling method. The lateral posterior method depth correction applied to the gamma camera method further underestimates the GFR ( Figs. 4 and 5 ).

ICC coefficient between the plasma sampling and gamma camera method of GFR estimation was 0.804 ( *p* -value < 0.05), it shows statistically significant good reliability (ICC between 0.75 and 0.90) between plasma sampling and gamma camera methods.

**Fig. 6:**
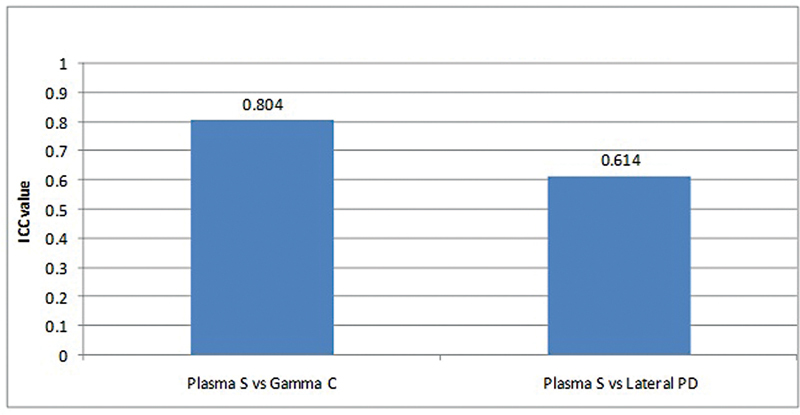
Intraclass correlation (ICC) analysis to study statistical reliability between plasma sampling with gamma camera method and plasma sampling with lateral posterior depth method of glomerular filtration rate (GFR) measurements.

ICC coefficient between the plasma sampling and lateral posterior method of depth correction for GFR estimation was 0.614 ( *p* -value < 0.05), it shows statistically significant moderate reliability (ICC between 0.50 and 0.75) between plasma sampling and LPD methods ( Table 3 , Fig. 6 ).

**Table 3:** Intraclass correlation (ICC) analysis to study statistical reliability by gamma camera method and lateral posterior depth method with reference to plasma sampling as a gold standard for GFR estimation

Methods	ICC coefficient	*p* -Value
Plasma sampling vs. gamma camera	0.804	0.001 a
Plasma sampling vs. lateral posterior depth	0.614	0.001 a

Abbreviation: GFR, glomerular filtration rate.

Note: *p* -Value by using ICC analysis. *p* -Value < 0.05 is considered to be statistically significantly reliable and vice versa.

a *p* -Value < 0.001.

**Fig. 7:**
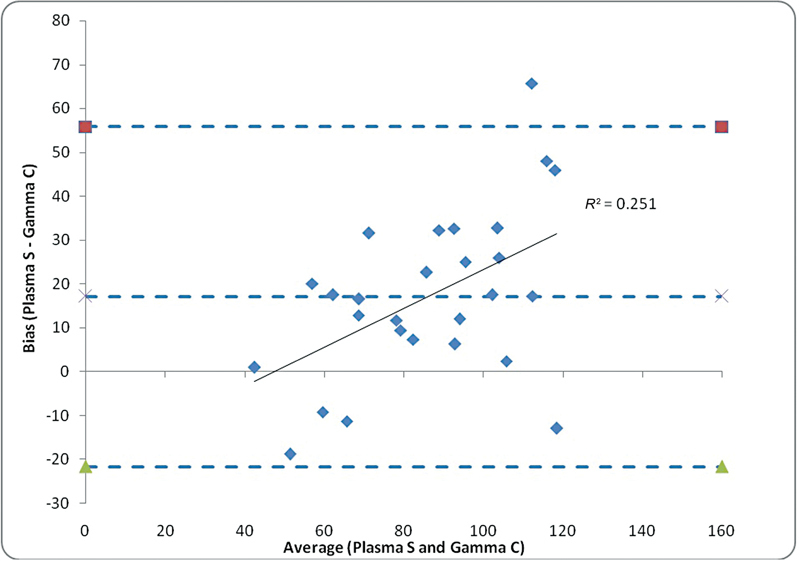
Bland–Altman plot for comparing the statistical agreement between plasma sampling (S) vs. gamma camera (C) method, the line of regression for predicting the bias (difference between plasma S and gamma C) using average of both the measurements is also shown in the figure.

**Fig. 8:**
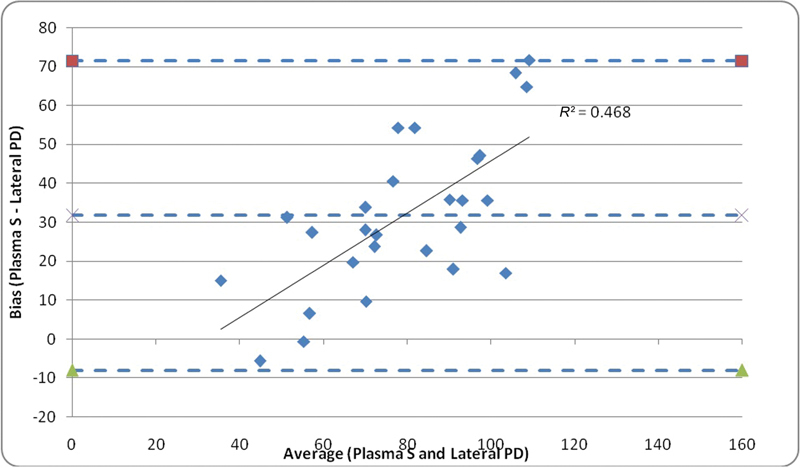
Bland–Altman plot for comparing the statistical agreement between plasma sampling (S) vs. lateral posterior depth (PD) method, the line of regression for predicting the bias (difference between plasma S and lateral PD) using average of both the measurements is also shown in the figure.

Bland–Altman analysis (average vs. difference or bias) was performed for comparing the agreement between plasma sampling versus gamma camera methods and plasma sampling versus lateral posterior method for depth correction, respectively, for GFR estimation. It is noted that the % *R* ^2^ value is minimum for plasma sampling versus gamma camera method (% *R* ^2^ value = 25.1%), which makes it the better method for calculating GFR as against plasma sampling versus lateral posterior method of depth correction (% *R* ^2^ value = 46.8%) ( Figs. 7 and 8 ).

**Fig. 9:**
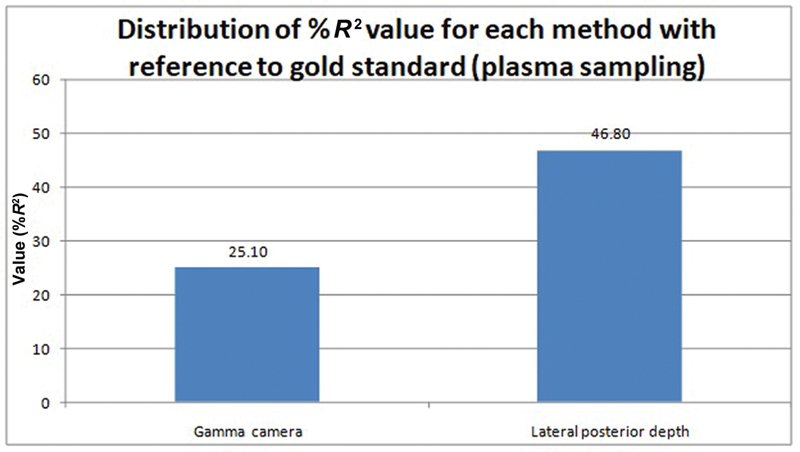
Distribution of % *R* ^2^ value for each method using line of regression for predicting the bias (difference) based on the average of both the measurements as a part of Bland–Altman analysis for comparing the statistical agreement between the methods. Lower % *R* ^2^ indicates better method and vice versa. Glomerular filtration rate (GFR) by plasma sampling is considered to be the gold standard.

It was concluded that there is no significant agreement between plasma sampling versus gamma camera method and plasma sampling versus lateral posterior method for depth correction for GFR measurements; however, the evidence of systemic bias is lower for the gamma camera method compared with the lateral posterior method for depth correction as against to plasma sampling method (shown in Fig. 7 in terms of % *R* ^2^ value) ( Table 4 ; Fig. 9 ).

**Table 4:** Linear regression analysis for the prediction of difference (bias) in GFR estimation by gamma camera method and lateral posterior depth method with reference to plasma sampling as a gold standard using average of both the measurements by using Bland–Altman analysis

	Value (mL/min) with reference to plasma sampling			Regression analysis for prediction of bias			
Method of GFR estimation	Mean bias [with reference to plasma sampling]	Lower limit of agreement	Upper limit of agreement	Standard beta coefficient	*p* -Value for testing Beta = 0	% *R* ^2^ value	Statistical evidence of systematic bias
Gamma camera	17.14	–21.60	55.88	0.502	0.008 a	25.1%	Evident
Lateral posterior depth	31.73	–7.97	71.43	0.685	0.001 b	46.8%	Strongly evident

Abbreviation: GFR, glomerular filtration rate.

Note: *p* -Value by regression analysis. *p* -Value < 0.05 is considered to be statistically significant. Average is used as an independent variable (Bland–Altman method).

a *p* -Value < 0.01.

b *p* -Value < 0.001.

Out of 27 cases studied, 14 cases (51.9%) had right kidney depth more than left kidney depth and 13 cases (48.1%) had right kidney depth less than left kidney depth.

## Discussion

There are several studies proving that GFR generated by gamma camera-based Gates method is not accurate and hence the need for a standardized GFR estimation method. 3 One of the reasons cited for inaccurate results is the lack of correction for depth of kidneys.

Earlier studies have used various equations for estimating depth of kidneys. Some publications concluded that Tonnesen's formula underestimated the renal depth. This in turn underestimated GFR by Gates method that used Tonnesen's formula for depth correction. 5 6 7 Also, some of the other formulae for kidney depth calculation faired better with specific ethnicity groups, for example, Li Q's formula for Chinese individuals and Taylor's formula for European and American population. 8 9

Renal depth measurement can be done using either the CT scan or lateral views of the kidneys. Based on the attenuation coefficient of Tc-99m in soft tissue of 0.153, even a 1-cm error in renal depth measurement will lead to a 14 to 16% error in the calculation of GFR. 10 11 This error in renal depth measurement is likely to happen in cases of hydronephrosis, renal calculi, and renal tumor.

The use of CT scan for renal depth exposes the patient to ionizing radiation. Dynamic contrast-enhanced magnetic resonance imaging is another study used for estimation of GFR. 12 13 The advantage being that there is no use of ionizing radiation. However, this method is not clinically validated. Further, it is necessary that we check the renal function before use of contrast.

We chose the lateral posterior method of depth correction for two reasons. First, patients with the abovementioned conditions of hydronephrosis, renal calculi, and renal tumors were not included in the study. The design of the study had thus eliminated the sources of error in renal depth measurement. Second, acquiring the lateral view while conducting a dynamic renal scintigraphy is simple and clinically feasible without additional radiation exposure.

Some studies have reported that the depth of the right kidney is more than that of the left kidney. 5 14 15 In our study, in 51.9% patients, right kidney depth is more than left and in a comparable number of patients, left kidney depth was more than right.

In a publication by Mantri et al, kidney depth calculated manually from the lateral view was incorporated in the Gates formula and the GFR thus obtained was compared with that obtained using the automated depth correction method. The manual method of depth correction using lateral view was found to be better of the two; however, there was no correlation done with the gold standard plasma sampling method. The right and left kidney mean depth by the lateral view method were found to be 7.2 ± 1.1 and 7.0 ± 1.2 cm, respectively. 16

In our study, the mean right and left kidney depths was found to be 4.87 ± 0.92 and 4.71 ± 1.08 cm, respectively. The only variable between the gamma camera method and the lateral posterior method for depth correction is the estimated kidney depth. The lateral posterior method for depth correction underestimated the GFR probably because it underestimated the kidney depth as compared with the gamma camera-based Gates method with its automated estimate of kidney depth. 8 17

This is a retrospective study that analyzed how the GFR by Gates method was affected by using the lateral posterior method for depth correction and whether the value so obtained correlated well with the gold standard plasma sampling method for GFR estimation.

The limitations of the study are its retrospective study design and smaller number of patients. We measured the skin to kidney center distance in the lateral view and posterior aspect 18 ; however, this does not take in to account the intrarenal attenuation.

## Conclusion

The lateral posterior method for depth correction while using the gamma camera-based Gates protocol is not a reliable method for depth correction in the western Indian adult population with preserved renal function and produces a greater bias in reference to the gold standard plasma sampling method.
